# Ethnic inequalities in ischemic stroke occurrence are partly mediated by air pollution: a Dutch nationwide follow-up analysis

**DOI:** 10.1186/s12889-026-27043-7

**Published:** 2026-03-16

**Authors:** Lieke van den Brekel, Ilonca Vaartjes, Diederick E. Grobbee, Gerard Hoek, Joreintje D. Mackenbach, Yvonne Koop

**Affiliations:** 1 Julius Center for Health Sciences and Primary Care, Utrecht University Medical Center, Utrecht University, P.O. Box 85500, Str 6.131, Utrecht, 3508 GA The Netherlands; 2 Institute for Risk Assessment Sciences, Utrecht University, Utrecht, The Netherlands; 3 Department of Epidemiology and Data Science, Amsterdam UMC, Vrije Universiteit Amsterdam, Amsterdam, The Netherlands; 4 Upstream Team, www.upstreamteam.nl, Amsterdam, The Netherlands; 5 Amsterdam Public Health Research Institute, Amsterdam, The Netherlands

**Keywords:** Migrant, Air pollution, Environmental justice, Climate justice

## Abstract

**Background:**

Ethnic disparities in ischemic stroke are substantial and differences in exposure to air pollution have been demonstrated as well. However, the role of air pollution in mediating ethnic disparities in risk of ischemic stroke remains unclear. Understanding this relationship is crucial, as air pollution is a major modifiable risk factor for ischemic stroke. This study aims to determine whether air pollution exposure mediates ischemic stroke risk differences between Indonesian, Surinamese, Dutch Caribbean, and ethnic Dutch populations.

**Methods:**

We carried out a nationwide cohort study in the Netherlands (2014–2019), including 9,248,484 residents aged 30 or older, free of ischemic stroke at baseline, of Dutch, Surinamese, Indonesian, or Dutch Caribbean ethnicity. Ethnicity was defined based on the country of birth of the individual and their parents. Air pollution exposure was measured via annual residential estimates of particulate matter < 2.5 micrometers (PM_2.5_) and nitrogen dioxide (NO_2_) for 2014. The study outcome was time to ischemic stroke occurrence or mortality, identified from nationwide hospital discharge and death registries using ICD-9/ICD-10 codes.

**Results:**

The cohort included 314,082 (3.4%) Indonesian, 204,158 (2.2%) Surinamese, 69,580 (0.8%) Dutch Caribbean and 8,660,664 (93.7%) ethnic Dutch individuals. Air pollution levels were highest among Surinamese and lowest among Dutch. For Surinamese, NO_2_ mediated 13.8% (11.0-17.5%) and PM_2.5_ mediated 7.9% (6.3–9.9%) of the ischemic stroke difference compared to ethnic Dutch. For Dutch Caribbeans, NO_2_ mediated 14.7% (9.8–26.7%) and PM_2.5_ mediated 8.0% (8.1–23.3%). Percentages could not be calculated for Indonesians.

**Conclusion:**

Air pollution substantially mediates the increased ischemic stroke risk in Surinamese and Dutch Caribbean populations. These findings emphasize the urgent need for air pollution reduction strategies to mitigate the disproportionate ischemic stroke burden in these populations.

**Supplementary Information:**

The online version contains supplementary material available at 10.1186/s12889-026-27043-7.

## Background

 Ischemic stroke (IS) remains a significant global public health problem, contributing to high morbidity and mortality worldwide [1]. The incidence of IS varies considerably across ethnic groups. In Western Europe, several minority ethnic populations have a similar or elevated risk of IS compared to majority populations [2]. Of the largest minority ethnic groups in the Netherlands, individuals of Surinamese origin have been shown to have a higher incidence of IS compared with the ethnic Dutch population. More modest elevations have been reported for individuals of Dutch Caribbean (e.g. originating from Aruba, Curaçao, Saint-Martin, Bonaire, Saint-Eustatius and Saba) and Indonesian origin [3]. Age-standardized IS incidence rates per 100,000 person-years have shown to be highest among Surinamese (280 [95%CI 263–298]), followed by Dutch Caribbean (220 [176–264]) and Indonesian (194 [186–202]) ethnic groups compared to ethnic Dutch (187 [186–188]) [3]. Suriname, Indonesia and the Dutch Caribbean are former Dutch colonies and the countries of origin of the 3^rd^, 4^th^ and 6^th^ largest minority ethnic populations in the Netherlands, respectively. The historical (post)colonial relationship between these countries and the Netherlands likely influences the social and health challenges currently faced by these communities [4, 5].

Ethnicity is a multidimensional social construct shaped by sociocultural factors such as shared history, cultural practices, and lived experiences, rather than a biological attribute [6, 7]. Structural determinants, including socioeconomic position (SEP), access to healthcare, discrimination, and environmental conditions, are embedded within broader sociopolitical contexts and influence intermediary factors such as residential environments and material resources [8]. Through these pathways, ethnicity may become associated with IS risk.

A major health concern that is disproportionally affecting minority ethnic populations is exposure to ambient air pollution [9]. Globally, including in the Netherlands, minoritized groups are exposed to higher levels of air pollution compared to majority populations [9, 10]. For instance, we previously demonstrated that populations with a Surinamese, Indonesian or Dutch Caribbean background had means residential concentrations of NO_2_ being 21.4%, 8.4% and 18.3% higher compared to Dutch, respectively. Residential PM_2.5_ concentrations were 2.3–4.1% higher than for ethnic Dutch. The burden of IS attributable to air pollution is high. In 2019, ambient particulate matter alone was estimated to have caused 215,000 disability adjusted life years and 137,000 deaths from IS in Western Europe [11]. The proposed pathological mechanisms of air pollution-induced IS include inflammation, oxidative stress and increased blood coagulation, resulting in endothelial dysfunction and atherosclerotic plaque progression [12, 13]. Furthermore, exposure to air pollution exacerbates traditional risk factors for IS such as diabetes, hypertension, atrial fibrillation and lipoprotein dysfunction [12, 14].

Despite clear evidence of inequalities in exposure to air pollution, the extent to which air pollution mediates the relationship between ethnicity and IS has not yet been quantified. Such quantification is essential to guide interventions aimed at improving equity in cardiovascular health and reducing the overall societal burden due to IS. Therefore, we addressed the following research question: To what extent are inequalities between Dutch, Indonesian, Surinamese and Dutch Caribbean populations in ischemic stroke occurrence mediated by exposure to air pollution?

## Methods

### Study population

**Fig. 1:**
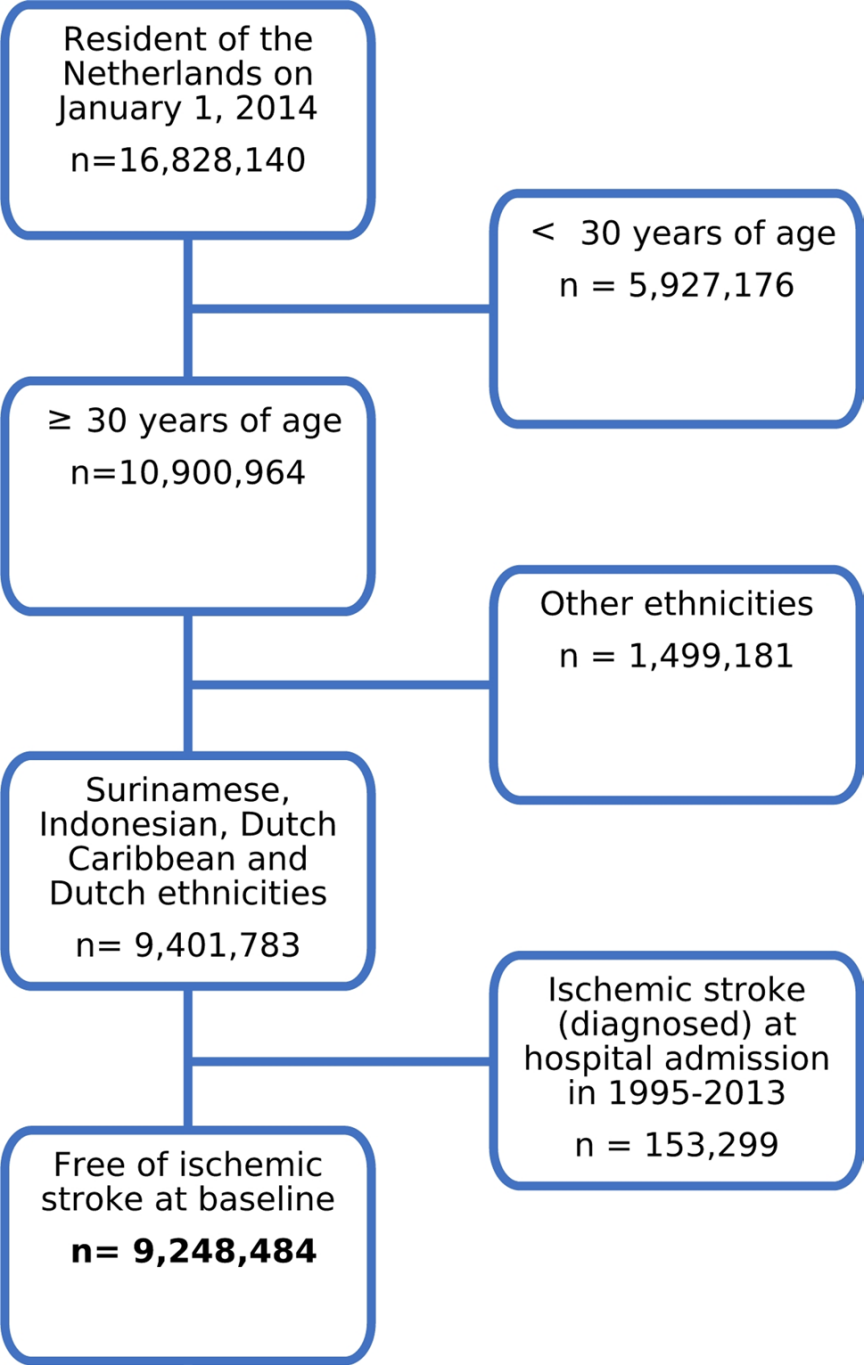
Flow chart of study population selection

For this longitudinal study between Jan 1, 2014 and Dec 31, 2019, we linked long-term home-address outdoor air pollution estimates to nationwide registries centralized by Statistics Netherlands (CBS). We selected from the national population registry all individuals ≥ 30 years of age and of Dutch, Surinamese, Indonesian or Dutch Caribbean backgrounds that were registered at an address in the Netherlands on Jan 1, 2014. We selected these country of origins as these are the largest ethnic populations in the Netherlands that have shown to have a higher incidence of ischemic stroke compared to ethnic Dutch. Individuals that were hospitalized with IS in 1995–2013 were excluded to create a cohort free of IS at baseline (*n* = 9,248,484 ). Information on medical history before 1995 was not available. In Fig. 1a flowchart illustrating the selection of the study population is provided.

### Outcome

The outcome studied was the incidence of IS, determined by either hospitalization with IS as the primary cause or mortality from IS. The information on in-hospital diagnosis originated from the nationwide hospital discharge registries harmonized by the Dutch Hospital Data foundation (DHD). The hospital discharge registries and cause of death registry were accessed through CBS. ICD-9 codes 434–436 and ICD-10 codes I63 and G45 were used to identify the IS events from the registries. ICD-9 and ICD-10 codes for the identification of stroke have been proven to be reliable in studies of patients with stroke [15–17]. See supplemental Table 1 for the ICD subcodes used to identify events.

### Determinant

Dutch, Surinamese, Indonesian and Dutch Caribbean groups were identified based on country of birth of the individual and their parents, according to Statistics Netherlands’ definition for migration background [18]. If both the individual and their parents were born in the Netherlands, ethnicity was determined as Dutch. If the individual was born abroad but both parents in the Netherlands, ethnicity was also determined as Dutch. If both the individual and their parents were born abroad, ethnicity was based on the country of birth of the individual. If the individual was born in the Netherlands but one or both parents abroad, ethnicity of the individual was determined based on the country of birth of their parent born abroad or in case of differing countries on the mothers’ country of birth.

### Mediators

The 2014 average concentrations of ambient NO_2_ and PM_2.5_ originated from nationwide maps created by the National Institute for Public Health and the Environment and the Dutch Nationaal Samenwerkingsprogramma luchtkwaliteit [19]. The maps are based on emission and dispersion information and have a resolution of 25 m. Model predictions and mapped absolute levels generally agree well with measurements for NO_2_ and PM_2.5_ [19, 20]. Home address concentrations were extracted by the Geoscience and Health Cohort Consortium and transferred into a quantitative dataset for uploading to CBS’ secure analysis environment [19]. The air pollutants were standardized by their interquartile range (IQR).

### Confounders

**Fig. 2:**
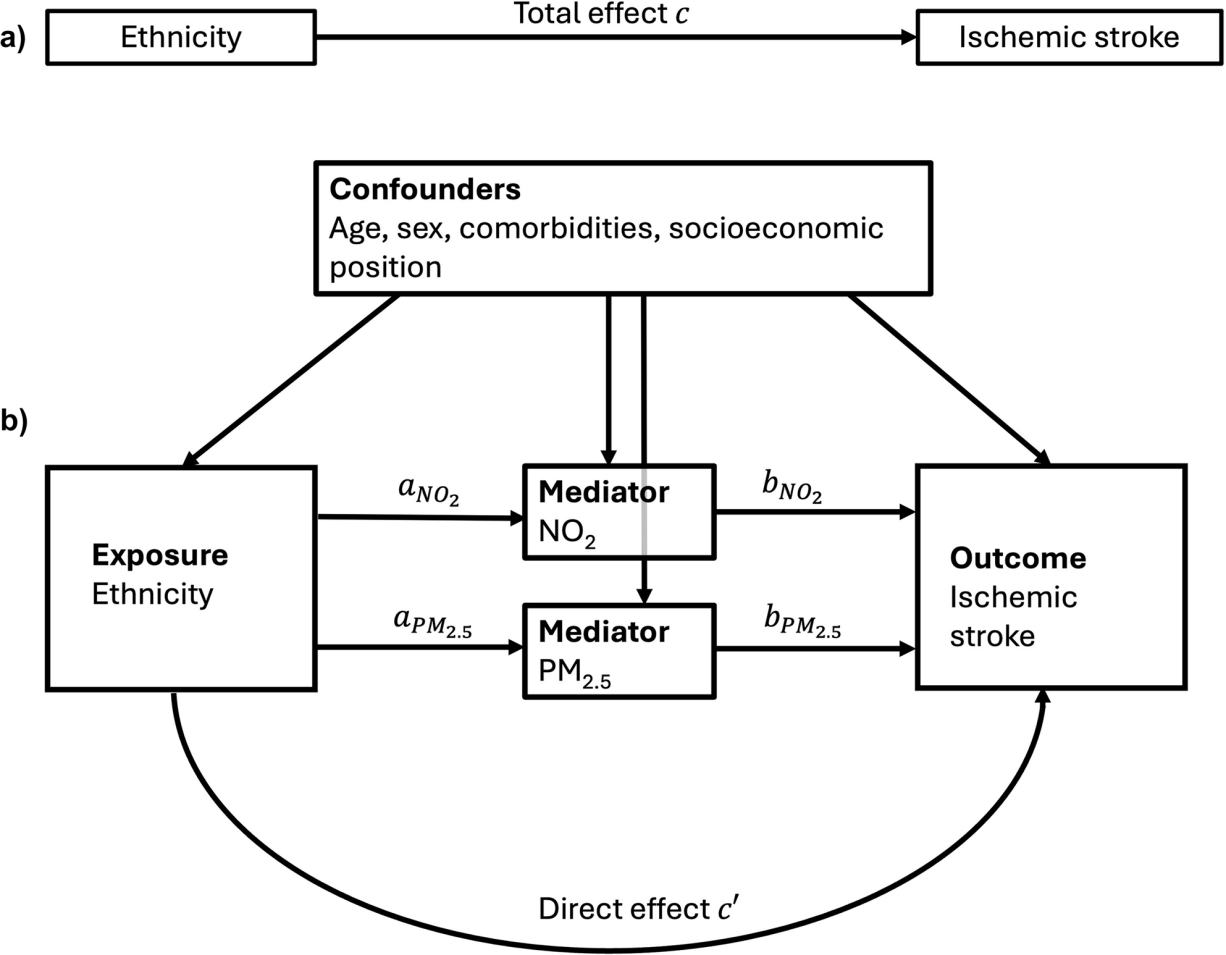
Directed acyclic graph of the relationship between (**a**) ethnicity and ischemic stroke and (**b**) mediated by PM_2.5_ and NO_2_, including confounders. NO_2_ = nitrogen dioxide, PM_2.5_ = particulate matter < 2.5 micrometers. Ethnicity included Surinamese, Indonesian and Dutch Caribbean ethnic groups compared to ethnic Dutch as reference

Based on the directed acyclic graph (DAG) in Fig. 2, several potential confounders were identified. Age in years and sex as registered at birth were retrieved from the national population register. To account for comorbidities and their underlying lifestyle factors, which may cluster within socially defined populations and potentially (non-causally) correlate with air pollution and IS, we adjusted for the Charlson Comorbidity Index. This index, calculated using the method developed and updated by Quan et al. was based on the ICD-9 and ICD-10 codes for 12 comorbidities recorded in hospital discharge registries between 1995 and 2013 [20]. This index has a range of 0 to 24 points, with higher scores reflecting a greater number and severity of comorbidities. Individual level SEP was approximated by a combined indicator of household disposable standardized income and taxable assets derived from the tax register. Household disposable income was standardized by household composition, according to the Statistics Netherlands method [21]. Taxable assets mainly consist of houses, shares and savings minus debts. Income and assets were standardized into percentiles, combined into a continuous indicator and subsequently divided into tertiles to indicate a low, middle or high SEP. Neighbourhood mean standardized household income in euros was used as an additional SEP indicator.

SEP occupies a complex position within the causal framework. SEP may act as a confounder, given its shared associations with ethnicity, air pollution exposure, and ischemic stroke risk. At the same time, SEP may partially lie on the causal pathway from ethnicity to ischemic stroke, as it could reflect downstream social and material consequences of structural disadvantage and discrimination. In addition, susceptibility to air pollution may vary by SEP, implying potential effect modification. Given these multiple plausible roles, we present both minimally adjusted models (adjusted for age and sex) and fully adjusted models (additionally adjusting for comorbidity burden and individual- and neighbourhood-level SEP). Minimally adjusted models are presented as the primary analyses.

For a sensitivity analysis, we added a regional indicator that divides the Netherlands into 40 adjacent areas, also known as COROP [COördinatie commissie Regionaal OnderzoeksProgramma] regions. These correspond to European Level 3 NUTS regions and were added to account for potential regional clustering.

### Data analysis

**Fig. 3:**
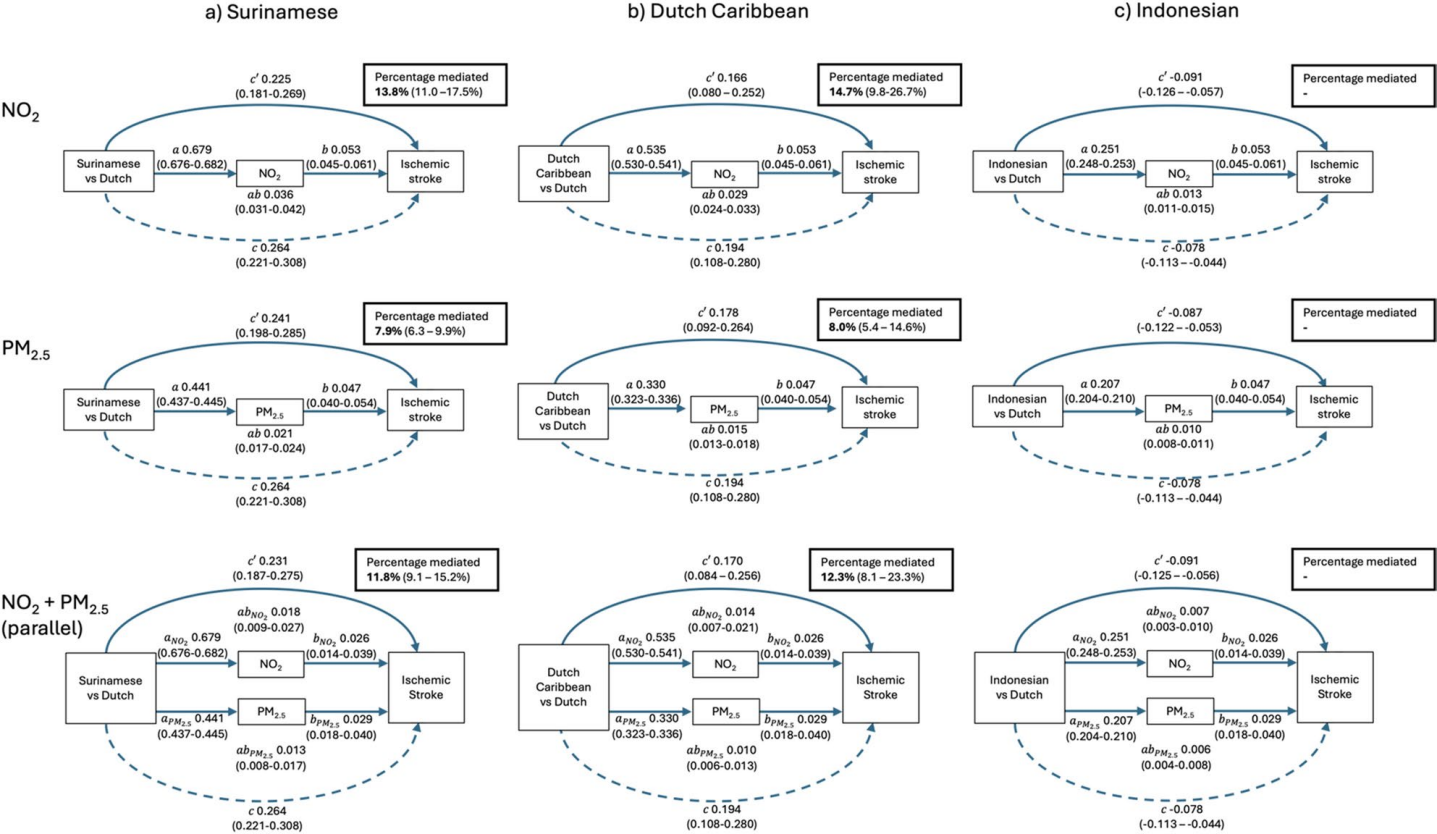
Mediation analysis of ischemic stroke differences relative to ethnic Dutch for (**a**) Surinamese (*n* = 204,158), (**b**) Dutch Caribbean (*n* = 69,580) and (**c**) Indonesian (*n* = 314,082) ethnic groups in the Netherlands. Models are adjusted for sex and age. Values represent *β*-coefficients (95% CI) from formal mediation analyses: *a*-paths indicate ethnic differences in air pollution exposure, *b*-paths the association between air pollution and ischemic stroke, *ab*-paths the indirect effect, *c*-paths the total effect, and *c’*-paths the direct effect. NO_2_= nitrogen dioxide, PM_2.5_ = particulate matter < 2.5 micrometers

Population characteristics and air pollution concentrations were presented as frequencies (%) for count data and means (SD) or medians (IQR) for continuous data. To assess the mediating effects of PM_2.5_ and NO_2_ on the relationship between ethnicity and IS incidence, we conducted two single mediation analyses and a parallel mediation analysis using MacKinnon’s product-of-coefficients method [22]. Linear regression was applied to the mediator models (*a*-paths, Fig. 3) to evaluate the associations between ethnicity and PM_2.5_ or NO_2_, standardized by their IQRs. Cox proportional hazard regression was used to estimate the total effect of ethnicity on time to IS (*c*-paths), the associations of PM_2.5_ or NO_2_ with IS (*b*-paths) and the direct effect (*c'*-paths). We performed two sets of mediation analyses: minimally adjusted models (adjusting for sex and age) and fully adjusted models (further accounting for the Charlson comorbidity index and SEP at the individual and neighbourhood level), as depicted in the DAG (Fig. 2). The minimally adjusted models were presented as the primary results, because comorbidities and SEP might act as both confounders and mediators. While these factors were expected to primarily influence the *b*-paths (air pollution–IS associations) and total effects (ethnicity–IS associations), they were included in the *a*-paths to satisfy mediation analysis requirements of the same confounders in all models. Since the determinant-mediator interactions were not statistically significant, $b$-paths were consistent across ethnic groups. We quantified the indirect effects of ethnicity on IS incidence through air pollution by multiplying the ${a}_{ij}$-paths and ${b}_{j}$-paths, producing indirect effect estimates denoted as ${ab}_{ij}$​, where $i$ represents the ethnic group (e.g., Surinamese vs. Dutch) and $j$ represents the air pollutant (e.g., PM_2.5_ or NO_2_). When the direct and indirect effects were in the same direction, the percentage mediated was calculated using:

$${{PM}_{ij}=ab}_{ij}\div\left({ab}_{ij}+{c}_{j}^{{\prime}}\right)\times100$$

To evaluate multicollinearity, Pearson correlation coefficients were calculated for PM_2.5_ and NO_2_, with a correlation threshold below 0.8 deemed appropriate for parallel mediation analysis. This analysis incorporated both air pollutants in models estimating the *b*-paths and *c’*-paths, with the combined percentage mediated calculated as:

$$\begin{aligned}{PM}_{i}&={(ab}_{i,{NO}_{2}}+{ab}_{i,{PM}_{2.5}})\\&\div{(ab}_{i,{NO}_{2}}+{ab}_{i,{PM}_{2.5}}+c{\prime})\times100\end{aligned}$$

Given the minimal amount of missing data (*n* < 66500 [< 1%]), individuals with incomplete information for any study variable were excluded from the analyses.

As a sensitivity analysis, spatial dependencies were addressed by incorporating a random intercept for COROP regions to the fully adjusted models. This random intercept was not included in the main analyses to avoid overadjustment, as ethnic differences in exposure are partly driven by residential patterns and spatial clustering in more polluted areas. Proportional hazards assumptions were validated using log-log cumulative hazard plots and graphical evaluations of Schoenfeld residuals, with no violations observed. The 95% confidence intervals for indirect effects and percentages mediated were computed via bootstrapping with 1,000 resamples, while Wald confidence intervals were used for other estimates. All analyses were conducted in R version 4.4.0 using multiple packages: the lmer function from lme4 [21], the glm function from stats and the coxph function from survival [23] were used for regression modeling, and the boot function from boot was used for bootstrapping.

### Ethical considerations

The study was conducted in compliance with Dutch privacy legislation. The medical ethical review board of the University Medical Center Utrecht provided a waiver for the Dutch Act on Medical Research Involving Human Subjects (no. 21–655/C).

## Results

The cohort comprised *n* = 9,248,484 individuals, including 314,082 (3.4%) of Indonesian background, 204,158 (2.2%) of Surinamese background, 69,580 (0.8%) of Dutch Caribbean background and 8,660,664 (93.7%) of Dutch background (Table 1). The Dutch Caribbean group had the lowest median (IQR) age (45.7 [37.0-55.9]) and Dutch had the highest (54.6 [43.9–66.6]. SEP was highest among ethnic Dutch. Comorbidities were most prevalent among ethnic Dutch, with 119,981 (1.4%) having a Charlson comorbidity index > 2. Over the six-year of follow-up period, 120,462 (1.3%) individuals developed ischemic stroke.

**Table 1:** Baseline characteristics stratified by ethnicity

	**Dutch Caribbean** (*n* = 69,580)	**Indonesian** (*n* = 314,082)	**Surinamese** (*n* = 204,158)	**Dutch** (*n* = 8,660,664)
Age in years– median (IQR)	45.7 (37.0, 55.9)	53.6 (44.2, 63.3)	48.3 (39.7, 57.8)	54.6 (43.9, 66.6)
Male – N (%)	34,020 (48.9%)	151,268 (48.2%)	92,615 (45.4%)	4,230,620 (48.8%)
SEP low – N (%)	35,388 (50.9%)	80,503 (25.6%)	87,466 (42.9%)	1,899,430 (21.9%)
SEP middle – N (%)	21,362 (30.7%)	109,297 (34.8%)	73,137 (35.8%)	3,059,305 (35.3%)
SEP high – N (%)	12,727 (18.3%)	124,252 (39.6%)	43,509 (21.3%)	3,701,668 (42.7%)
SEP missing – N (%)	103 (0.1%)	30 (< 0.1%)	46 (< 0.1%)	261 (< 0.1%)
Neighborhood average income in euros (**÷** 1000**)** – Median (IQR)	20.9 (18.0, 24.0)	23.3 (20.5, 26.4)	21.2 (17.9, 24.5)	22.7 (20.5, 25.2)
Neighborhood income missing – N (%)	265 (0.4%)	1,257 (0.4%)	369 (0.2%)	57,009 (0.7%)
Charlson comorbidity index > 2 - N (%)	672 (1.0%)	3,571 (1.1%)	1,992 (1.0%)	119,981 (1.4%)
Developed IS – N (%)	525 (0.8%)	3,376 (1.1%)	2,081 (1.0%)	114,480 (1.3%)
NO_2_ in$\mu$g/m^3^– Mean (SD)	23.2 (5.4)	21.2 (4.8)	24.2 (5.2)	19.5 (4.9)
PM_2.5_ in$\mu$g/m^3^– Mean (SD)	13.8 (1.3)	13.5 (1.3)	14.0 (1.1)	13.2 (1.4)
Missing air pollution – N (%)	972 (1.4%)	2,365 (0.8%)	1,818 (0.9%)	60,895 (0.7%)

*IQR* interquartile range, *SEP* socioeconomic position, *SD* standard deviation, *IS* ischemic stroke, *NO*_2_ nitrogen dioxide, *PM*_2.5_ particulate matter < 2.5 micrometers

Mean (SD) home-address concentrations of NO_2_ in $\mu$g/m^3^ were lowest among Dutch (19.5 [4.9]) and highest among Surinamese (24.2 [5.2]). PM_2.5_ concentrations were also lowest among Dutch (13.2 [1.4]) and highest among Surinamese (14.0 [1.1]).

Age- and sex-adjusted risk for IS was highest among Surinamese, with a hazard ratio (HR) of 1.30 (1.25–1.36) compared to ethnic Dutch. The HR for IS was 1.21 (1.11–1.32) for Dutch Caribbeans compared to ethnic Dutch. Indonesians had a lower risk of IS compared to Dutch (HR 0.92 [0.89–0.96]).

## Mediation analysis

In the models adjusted for age and sex, the $a$-paths from the single air-pollutant mediation analyses showed higher air pollution exposure among minority ethnic groups (Fig. 3), which is consistent with the crude home-address concentrations that are reported in Table 1. The highest exposure levels were observed for the Surinamese group, with a mean difference for NO_2_ exposure relative to the Dutch group of 0.679 (0.676–0.682), and for PM_2.5_ it was 0.441 (0.437–0.445).

Higher levels of air pollution were associated with an increased risk of IS, as indicated by the $b$-paths. The $\beta$-coefficient (log HRs) of stroke corresponding to an IQR (6.85 $\mu$g/m^3^) increase in NO_2_ was 0.053 (0.045–0.061) and for an IQR (1.66 $\mu$g/m^3^) increase in PM_2.5_ it was 0.047 (0.040–0.054) (Fig. 3), corresponding to HRs of 1.054 (1.046–1.063) and 1.048 (1.041–1.055), respectively. The indirect effects ($ab$-paths) of ethnicity on IS through NO_2_ or PM_2.5_ ranged from 0.010 (0.008–0.011) (Indonesians for PM_2.5_) to 0.036 (0.031–0.042) (Surinamese for NO_2_).

For Surinamese individuals, NO_2_ mediated 13.8% (11.0-17.5%) and PM_2.5_ mediated 7.9% (6.3–9.9%) of the disparity in IS incidence compared to Dutch individuals. Among Dutch Caribbean individuals, NO_2_ mediated 14.7% (9.8–26.7%) and PM_2.5_ mediated 8.0% (8.1–23.3%) of the difference. For Indonesians, direct and indirect effects operated in opposite directions, preventing the calculation of meaningful percentages mediated by air pollution.

Pearsons’ correlation coefficient between NO_2_ and PM_2.5_ was 0.789. In the parallel mediation model, the $\beta$-coefficients (log hazard ratios) of NO_2_ and PM_2.5_ on ischemic stroke ($b$-paths) decreased compared to the single-pollutant mediation, with the most substantial reduction observed for NO_2_. The resulting percentages mediated were 11.8% (9.1–15.2%) for Surinamese and 12.3% (8.1–23.3%) for Dutch Caribbean individuals. These are lower than the sum of the percentages mediated from single-pollutant mediation analyses, demonstrating a partial offsetting effect between the pollutants.

Further adjustment for the Charlson comorbidity index and SEP led to reductions in the c-paths and c’-paths, with minimal increases in the ab-paths (Figure S2). This adjustment resulted in higher percentages mediated compared to the minimally adjusted models. For the difference in IS incidence between Surinamese and Dutch individuals, NO_2_ mediated 20.8% (15.7–27.9%), and PM_2.5_ mediated 14.1% (10.8–18.8%). For the difference between Dutch Caribbean and Dutch individuals, the percentages mediated were 27.5% (14.3–100.0%) by NO_2_ and 18.2% (9.7–84.2%) by PM_2.5_.

### Sensitivity analysis

Including a random intercept for COROP regions in the fully adjusted analyses markedly reduced the $a$-paths, though they remained statistically significant (Figure S3). The lowest $\beta$-coefficients for PM_2.5_ exposure were observed among Indonesians (0.069 [0.068–0.070]) and the highest for NO_2_ exposure in Surinamese (0.214 [0.212–0.216]) compared to Dutch individuals. All $b$-paths and *ab*-paths also decreased but remained statistically significant in the single-air pollutant models. Among Surinamese, percentages mediated declined to 3.1% (1.5–5.2%) for NO_2_ and 3.4% (1.6–5.9%) for PM_2.5_. Among Dutch Caribbeans, percentages mediated were reduced to 5.4% (1.7–25.5%) for NO_2_ and 5.0% (1.6–24.4%) for PM_2.5_.

## Discussion

Our mediation analysis, conducted in a nationwide cohort using a longitudinal design, demonstrates that a substantial portion of the higher incidence of IS among Surinamese and Dutch Caribbean ethnic populations in the Netherlands, compared to ethnic Dutch populations, can be attributed to higher exposure to air pollution. Specifically, NO_2_ mediated up to 14.7% of the difference in IS risk and PM_2.5_ mediated up to 8.0%. In the parallel mediation models, the percentage mediated was higher than in the PM_2.5_-only model, but lower than in the NO_2_-only model, suggesting a partial offsetting effect between pollutants. Despite higher air pollution exposure among Indonesians compared to ethnic Dutch, no corresponding increase in IS risk was observed.

To our knowledge, this is the first study to investigate the mediating role of air pollution in ethnic disparities in cardiovascular disease outcomes. Of the most comparable studies, a British study identified air pollution as a mediator in the association between neighborhood deprivation and systolic blood pressure and two studies from the United States (US) Multi-Ethnic Study of Atherosclerosis (MESA) [24]. One of the MESA studies found that exposure to air pollution does not explain ethnic differences in carotid intima-media thickness [25]. The other study found that PM_2.5_ and O_3_ exposure contributed to ethnic disparities in hypertension [26]. Our findings for the *a*-path align with existing literature demonstrating disproportionate exposure to PM_2.5_ and NO₂ among minority ethnic groups, including those included in this study [9, 10]. For the *b*-paths, our observed log-HRs are consistent with effect estimates reported in prior population-based studies and meta-analyses on the association between stroke and exposure to NO_2_ and PM_2.5_ [27].

In addition to mediation through differential exposure, differential vulnerability to air pollution may also contribute to ethnic inequalities in ischemic stroke. Such heterogeneity might arise from variation in susceptibility to harmful exposures, shaped by factors such as chronic psychosocial stress, limited access to material and healthcare resources, and broader socioeconomic constraints [28–31]. Differential effects of air pollution on cardiovascular disease outcomes by ethnicity have been documented previously, including in our own work, and should be considered complementary to the current findings [32, 33].

Additional adjustment for the Charlson comorbidity index and SEP lowered the proportions mediated in our study. We attribute this mainly to the mediating role of these covariates in the relationship between ethnicity and IS, lowering the *c-*paths and *c’*-paths, in addition to their confounding role. This is supported by a mediation analysis from the REGARDS study finding that over 40% of the increased stroke risk among Black populations in the US was mediated by traditional risk factors (e.g., atrial fibrillation, diabetes, hypertension, and smoking), rising to 50% with the inclusion of SEP indicators [34] Another mediation analyses of the REGARDS study on Black and White differences in hypertension found that 33% of the differences were mediated by social determinants of health including education, income, living in disadvantaged neighbourhoods or a rural area, social connections and public health infrastructure [35].

We included a random intercept for COROP regions to account for spatial clustering, initially expecting it to primarily influence standard errors. However, this adjustment had a substantial impact on the effect estimates. The reduction observed in the *a*-path likely reflects overadjustment, as exposure inequalities are largely driven by the clustering of ethnic Dutch populations in lower-pollution areas and minority ethnic groups in higher-pollution areas [9, 36]. For the *b*-path, part of the reduction may result from the random intercept for COROP regions indirectly addressing residual confounding, although the specific contributing factors remain unclear. Lifestyle factors such as smoking and physical activity—both established risk factors for IS—may cluster in high-pollution areas, potentially making air pollution a proxy for these behaviors [37]. However, evidence from a Danish study indicates that adjusting for lifestyle variables leaves HRs for air pollution and cardiovascular disease unchanged or slightly increased [38]. Future research requires diverse population data on lifestyle habits to clarify their confounding role in air pollution–IS associations across ethnic groups.

We do not have an explanation for the lower risk of IS among Indonesians compared to ethnic Dutch, other than the possibility that disease risk in healthier immigrant populations may gradually align with that of the majority population [39]. An earlier study reported a higher IS risk among Indonesians relative to Dutch, but they used data between 1998 and 2010 [3]. There is currently a lack of trend data on cardiovascular disease risk factors and outcomes across minority ethnic groups in the Netherlands, including Indonesians, which is essential to identify key risk and protective factors.

A strength of this study is its use of nationwide data, providing a large sample to detect the relatively small effect estimates of air pollution across several ethnic populations in the Netherlands. The parallel mediation models allowed us to assess the joint effects of NO_2_ and PM_2.5_, providing a more nuanced understanding of their respective contributions to stroke risk. The largest limitation of this study is the lack of data on potential confounders. While we addressed this indirectly with a regional indicator, additional confounder information, including traditional cardiovascular disease risk factors, is needed for future studies. Additionally, there is ethnic, cultural and ancestral diversity as well as potential variation in (risk factors for) IS within the Surinamese and Dutch Caribbean groups that we were unable to take into account [40]. Furthermore, defining ethnic groups based on country of origin does not capture ethnic populations that are not defined by first- or second-generation migration status, such as later-generation immigrants or ethnic minorities within countries of origin (e.g. Roma populations). Income and taxable assets are incomplete measures of socioeconomic position. However, in a previous study using similar data, we observed only minor differences in exposure estimates when applying a more comprehensive composite socioeconomic index [9]. Ascertainment of stroke using ICD codes from hospital discharge registries may lead to some underestimation, as strokes occurring without hospital presentation or death, and TIAs not presenting to in-hospital care, are not captured. However, most individuals with stroke events in the Netherlands present to a hospital. Prior studies report positive predictive values of 94–97% for vascular outcomes including stroke, using hospital discharge registries [41, 42]. Exposure was assessed at a single time point (2014), precluding assessment of changes during follow-up or lifetime exposure. Nevertheless, spatial contrasts in air pollution in the Netherlands are relatively stable over time [43–45] and prior sensitivity analyses restricted to individuals who had lived at the same address for at least five years showed similar exposure–disease associations [32].

The finding that preventable disparities in air pollution exposure account for a significant portion of ethnic differences in IS occurrence highlights the critical importance of implementing air pollution reduction policies. Addressing structural factors, including systemic racism that shapes residential patterns and neighborhood quality, is essential for mitigating broader health inequities linked to environmental exposures [28, 29, 46, 47]. Future studies should examine the clustering of ethnic groups along with the distribution of both adverse and protective environmental factors, including air pollution, and subsequent cardiovascular risk factors to inform targeted interventions. The lower IS incidence among Indonesians emphasizes the need to study trends in cardiovascular disease risk across diverse populations.

## Conclusion

Up to 13.8% of the elevated IS risk among Surinamese and Dutch Caribbean populations, compared to ethnic Dutch, is mediated by air pollution exposure. These results underscore the critical need for air pollution reduction strategies to mitigate the disproportionate IS burden in these populations.

## Supplementary Information

Supplementary Material 1.

## Data Availability

Under certain conditions, the underlying encrypted microdata are accessible from Statistics Netherlands. For further information: microdata@cbs.nl. If verification of the analyses is desired and Statistics Netherlands provides access to the microdata, we will provide the R-scripts for cohort-building and analyses, upon reasonable request. The geo-data can be requested from the Geoscience and Health Cohort Consortium (GECCO). More information can be found at [http://www.gecco.nl/](http:/www.gecco.nl) .

## Abbreviations

CBS: Statistics Netherlands [Centraal Bureau voor de Statistiek
COROP: regional indicator dividing the Netherlands in 40 adjacent regions [coordinatiecommissie regional onderzoeksprogramma
HR: hazard ratio
ICD: International Classification of Diseases
IQR: interquartile range
IS: ischemic stroke
NO_2_: nitrogen dioxide
NUTS: nomenclature of territorial units for statistics (Nomenclature des Unités territoriales statistiques)
PM_2.5_: particulate matter < 2.5 micrometers
SD: standard deviation
SEP: socioeconomic position
US: United States
