# Supplementary material for: Ethnic inequalities in ischemic stroke occurrence are partly mediated by air pollution: a Dutch nationwide follow-up analysis
Source: BMC Public Health. 2026 Mar 16;26:1390. doi: 10.1186/s12889-026-27043-7 (PMC13122870; doi:10.1186/s12889-026-27043-7)
Supplement: Supplementary file 1 — Supplementary Material 1. [file 12889_2026_27043_MOESM1_ESM.docx]

# Supplemental material - Ethnic inequalities in ischemic stroke are partly mediated by air pollution: a Dutch nationwide follow-up analysis

L. van den Brekel et al.

Table S1. ICD-9 and ICD-10 subcodes used for defining the outcome and history of IS

Figure S2. Fully adjusted mediation analysis of ethnic differences in ischemic stroke

Figure S3. Sensitivity analysis including a random intercept for COROP regions in the fully adjusted models

| Table S1. ICD-9 and ICD-10 subcodes used for defining the outcome and history of IS | | |
| --- | --- | --- |
| ICD-9 and ICD-10 codes | **Categorize as developed IS if this ICD code is registered during follow up*** | **Exclude people with this ICD code in history **** |
| IS ICD-10 | | |
| I63.0-I63.9 Cerebral infarction | + (IS) | + (IS) |
| I64 Stroke, not specified as haemorrhage or infarction | - | + (IS) |
| G45.0-G45.9 Transient cerebral ischaemic attacs and related syndromes (TIA) | + (IS) | + (IS) |
| IS ICD-9 | | |
| 434.0-434.9 Occlusion of cerebral arteries | N/A | + (IS) |
| 435.0-435.9 Transient cerebral ischemia (TIA) | N/A | + (IS) |
| 436 Acute, but ill-defined, cerebrovascular disease | N/A | + (IS) |

**Figure S2**. Mediation analysis of ischemic stroke differences relative to ethnic Dutch based on fully adjusted models for a) Surinamese (n=204,158), b) Dutch Caribbean (n=69,580) and c) Indonesian (n=314,082) ethnic groups in the Netherlands.

**
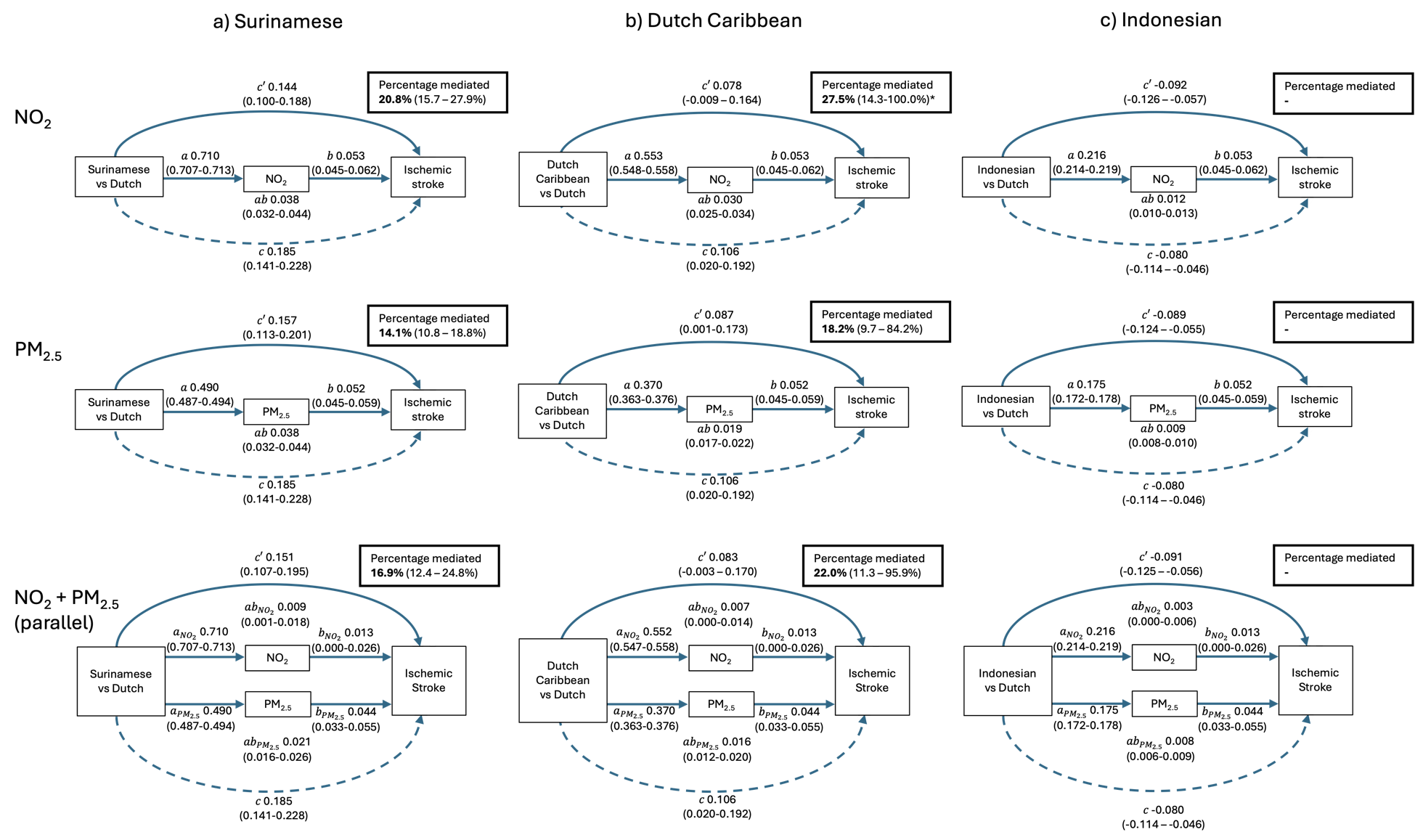
**

Fully adjusted models are adjusted for sex, age, Charslon comorbidity index and socioeconomic position. Values represent *β*-coefficients (95% CI) from formal mediation analyses: *a*-paths indicate ethnic differences in air pollution exposure, *b*-paths the association between air pollution and ischemic stroke, *ab*-paths the indirect effect, *c*-paths the total effect, and *c'*-paths the direct effect. *The upper bound of the bootstrapped 95% confidence interval is truncated at 100, NO_2_= nitrogen dioxide, PM_2.5_ = particulate matter <2.5 micrometers

**Figure S3**. Sensitivity analysis including a random intercept for COROP regions in the fully adjusted models. Mediation analysis of ischemic stroke differences relative to ethnic Dutch for a) Surinamese (n=204,158), b) Dutch Caribbean (n=69,580) and c) Indonesian (n=314,082) ethnic groups in the Netherlands


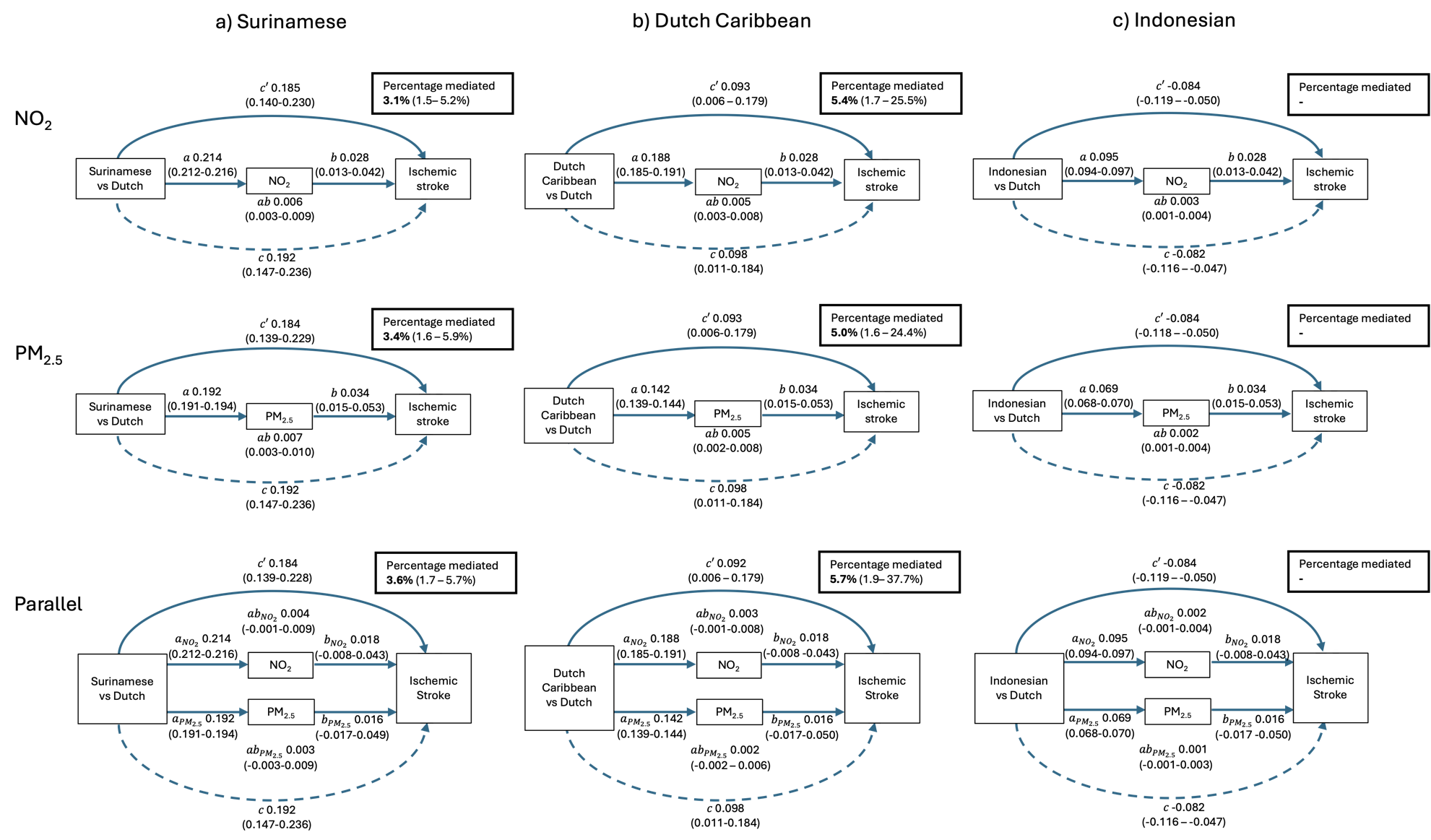


Fully adjusted models are adjusted for sex, age, Charslon comorbidity index and socioeconomic position. Values represent *β*-coefficients (95% CI) from formal mediation analyses: *a*-paths indicate ethnic differences in air pollution exposure, *b*-paths the association between air pollution and ischemic stroke, *ab*-paths the indirect effect, *c*-paths the total effect, and *c'*-paths the direct effect. COROP=regional indicator dividing the Netherlands in 40 adjacent clusters of municipalities, NO_2_= nitrogen dioxide, PM_2.5_ = particulate matter <2.5 micrometers
